# Skin Dose Reduction by Layer-Stacking Irradiation in Carbon Ion Radiotherapy for Parotid Tumors

**DOI:** 10.3389/fonc.2020.01396

**Published:** 2020-08-14

**Authors:** Nobuteru Kubo, Yoshiki Kubota, Takahiro Oike, Hidemasa Kawamura, Makoto Sakai, Ayaka Imamura, Shuichiro Komatsu, Yuhei Miyasaka, Hiro Sato, Atsushi Musha, Naoko Okano, Katsuyuki Shirai, Jun-ichi Saitoh, Kazuaki Chikamatsu, Tatsuya Ohno

**Affiliations:** ^1^Gunma University Heavy Ion Medical Center, Maebashi, Japan; ^2^Department of Radiology, Saitama Medical Center, Jichi Medical University, Omiya-Ku, Japan; ^3^Department of Radiation Oncology, Faculty of Medicine, University of Toyama, Toyama, Japan; ^4^Department of Otolaryngology-Head and Neck Surgery, Gunma University Graduate School of Medicine, Maebashi, Japan

**Keywords:** carbon ion radiotherapy, head and neck tumors, layer-stacking irradiation, parotid tumors, radiation dermatitis, dose surface-area histogram, skin dose

## Abstract

**Background:** Layer-stacking irradiation (LSI) results in the accumulation of multiple small spread-out Bragg peaks along the beam direction. Although the superiority of LSI to conventional passive irradiation (CPI) regarding normal tissue sparing is theoretically evident, the clinical benefit of LSI has not been demonstrated. Here, we compared LSI with CPI using the same treatment planning-computed tomography images used for carbon ion radiotherapy (CIRT).

**Methods:** Twenty-one parotid tumors were analyzed. The clinical target volume (CTV) 1 and CTV2 encompassed the parotid grand and the tumor, respectively. CTV1 and CTV2 received 36 Gy (RBE: relative biological effectiveness) in nine fractions and 64 Gy (RBE) in 16 fractions, respectively, using either LSI or CPI. CTV coverage was assessed by DX%, which is the dose covering at least X% of the target volume. Skin dose was assessed by SX, which is the skin surface area receiving at least X Gy (RBE).

**Results:** For CTV1 and CTV2, there were no significant differences in D2% between LSI and CPI. D50% and D98% were slightly higher for CPI; however, the absolute difference between the two methods was <3%. S10–S60 (in increments of 10) were significantly lower for LSI than for CPI (*P* < 0.001 for all parameters). LSI was associated with a significant trend toward dose reduction at the skin area irradiated with a higher dose by CPI (*P* < 0.001).

**Conclusions:** LSI achieved better skin sparing than CPI without sacrificing target volume coverage in parotid tumor patients.

## Introduction

**Figure 1:**
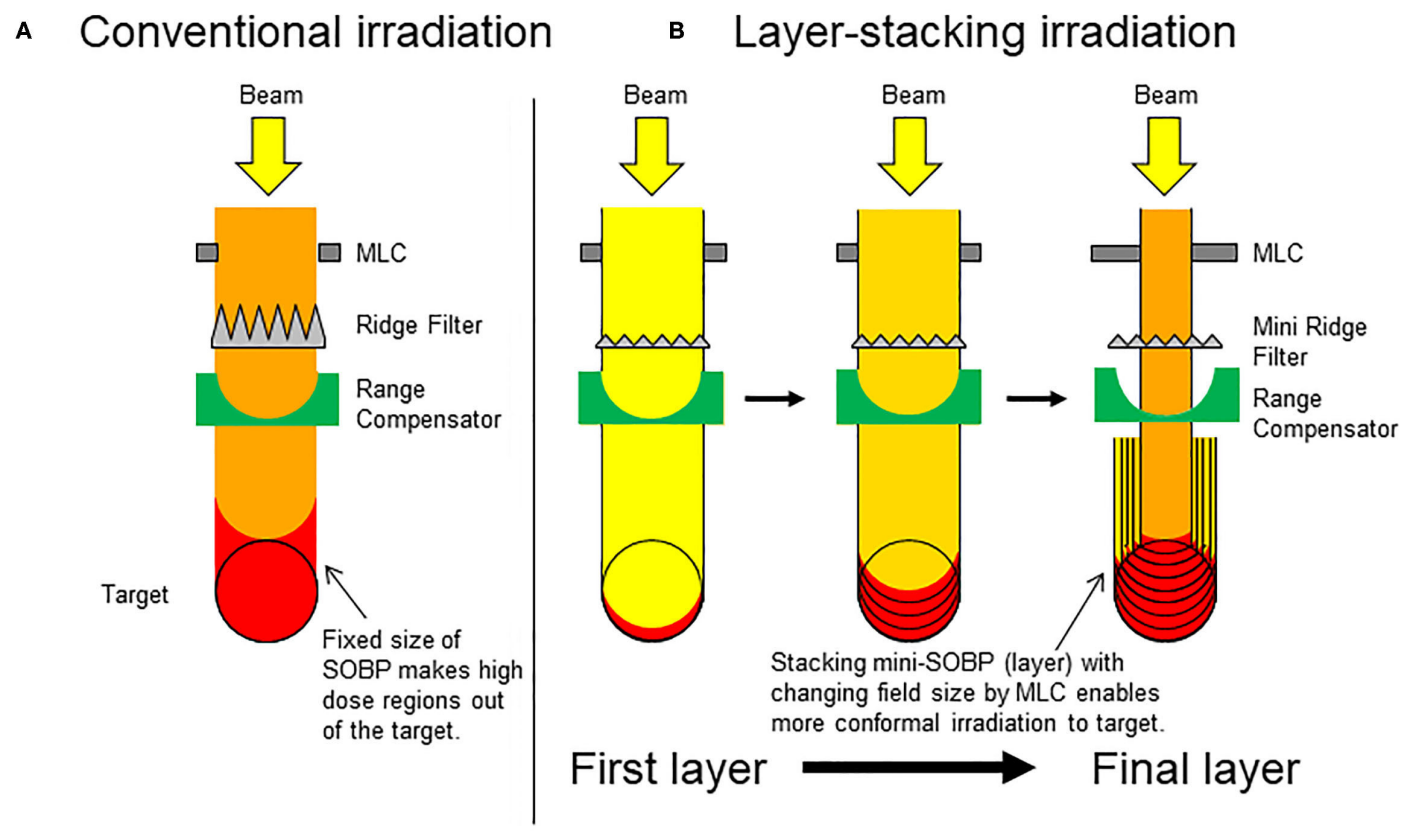
Schematic presentation of conventional passive irradiation and layer-stacking irradiation. **(A)** Conventional passive irradiation; **(B)** layer-stacking irradiation.

Carbon ion radiotherapy holds great promise in cancer treatment. Current evidence suggests that carbon ion radiotherapy is more effective for tumor control than standard care (1). In conventional passive irradiation (CPI) with carbon ions, treatment beams are broadened in the lateral direction using a pair of wobbler magnets and a scatterer, and the Bragg peaks are broadened along the beam direction using a ridge filter to form a spread-out Bragg peak (SOBP) (2). This enables dose distribution that is highly conformal to tumors. However, CPI methods have several shortcomings: i.e., normal tissues located at the entrance of the target receive excessive doses because the SOBP length is fixed by the diameter of the target (Figure 1A). This effect becomes greater in bulky tumors irradiated using long-length SOBPs, which increase the risk of toxicity to normal tissues. To overcome this issue, layer-stacking irradiation (LSI) was developed (3). In LSI, a finite number of small SOBPs are accumulated along the beam direction, contributing to dose reduction to normal tissues at the region near the entrance (Figure 1B). New carbon ion radiotherapy facilities prefer to adopt the spot-scanning technique, which is another irradiation method aimed at achieving high-dose conformation, However, already existing carbon ion radiotherapy facilities still employ passive beam treatment rooms, which are not adapted for spot-scanning. In Japan, about half of carbon ion radiotherapy facilities have passive beam treatment rooms. Therefore, LSI has the advantage that it can be used as an alternative method in facilities where the installation of scanning beam systems is prohibitive (4–6).

From these perspectives, the usefulness of LSI is theoretically evident, especially for the treatment of superficial tumors. However, the clinical benefit of this method over CPI remains to be demonstrated. To address this issue, we chose parotid tumors as a model in the present analysis. In carbon ion radiotherapy for parotid tumors, sparing of the skin is important because parotid glands are anatomically adjacent to the skin. A study that reported the outcomes of carbon ion radiotherapy for parotid tumors showed that the doses prescribed to the target were compromised in 57% of the patients to avoid exposure of the skin or the brain to high-dose irradiation (7). Another multi-institutional study that reported the outcomes of carbon ion radiotherapy for salivary gland tumors, 84% of which were parotid tumors, showed that the incidence of grade-3 dermatitis was 10% according to Common Terminology Criteria for Adverse Events version 4.0 (8). Based on these data, the present study compared treatment plans created using LSI with those created using CPI in the same set of patients with parotid tumors treated with carbon ion radiotherapy by analyzing target volume coverage and skin doses.

## Materials and Methods

### Patient Characteristics

Between October 2010 and March 2019, 21 consecutive patients with parotid tumors were treated with carbon ion radiotherapy at Gunma University Heavy Ion Medical Center (GHMC). Table 1 shows patient and tumor characteristics.

**Table 1:** Patient and tumor characteristics.

**Variable**	***n* (%)**
**Age**	
Median (range)	62 (42–87)
**Gender**	
Male	11 (52)
Female	10 (48)
**Histology**	
Adenoid cystic carcinoma	5 (24)
Adenocarcinoma	4 (19)
Mucoepidermoid carcinoma	3 (14)
Epithelial-myoepithelial carcinoma	3 (14)
Salivary duct carcinoma	3 (14)
Acinic cell carcinoma	1 (5)
Synovial sarcoma	1 (5)
Carcinoma	1 (5)
**T stage**	
T1	1 (5)
T2	2 (10)
T3	3 (14)
T4a	9 (43)
T4b	6 (29)
***N*** **stage**	
0	18 (86)
1	1 (5)
2	2 (10)
3	0 (0)
**M stage**	
0	21 (100)
1	0 (0)
**Primary or recurrent tumor**	
Primary tumor	15 (71)
Recurrence after surgery	6 (29)
**CTV volume (cm**^**3**^**)**	
CTV1 median (range)	83.0 (15.5–253.7)
CTV2 median (range)	62.7 (8.7–189.0)

### Treatment Planning

Computed tomography images used for treatment planning were acquired at 2-mm slice thickness. The voxel dimensions of all CT images were ~0.88 × 0.88 × 2.0 mm. Treatment plans were generated using XiO-N systems (Elekta, Stockholm, Sweden). Target volumes used in clinical practice were used in this study as follows: clinical target volume (CTV) 1 generally encompassed the whole anatomical site of the tumor origin (i.e., parotid grand), whereas CTV2 encompassed the tumor.

In the treatment planning for carbon ion radiotherapy, the unit Gy (RBE, relative biological effectiveness) is used to describe the prescribed dose (9). Thirty-six Gy (RBE) in nine fractions and 64 Gy (RBE) in 16 fractions were prescribed to CTV1 and CTV2, respectively.

Treatment plans using CPI were generated as described previously (10). The SOBP size used for CPI varied by 5 and 10 mm for horizontal and vertical beams, respectively.

In LSI, 5-mm SOBPs were stacked in the beam direction in steps of 2.5 mm using the range shifter, and the shape of multi leaf collimator (MLC) was changed at every step after 12 steps (i.e., 30 mm). The SOBP size varied by 2.5 mm. The initial shapes used for LSI were those used for conventional irradiation.

The same planning settings were used for CPI and LSI (e.g., the settings for proximal and distal margins to the targets, beam energy, and the number and direction of the beams), and the SOBP size was determined based on the target and proximal and distal margins.

### Plan Evaluation

Correlation analysis of carbon ion doses with CTVs or with the skin was performed using MIM Maestro (version 6.8.7., MIM Software Inc., Cleveland, OH, USA). D2%, D50%, D98%, and the homogeneity index (HI) were used as the endpoints for CTV coverage. DX% indicates the dose that covers at least X% of a given target volume. HI is calculated using the following equation: HI = (D2% – D98%)/D50% (11).

The skin volume was defined as the region within 0.02 cm under the skin surface (12). Skin surface area (cm^2^) was defined as the skin volume divided by 0.02. SX was used as the endpoint for dose-skin surface area analysis, where SX indicates the skin surface area irradiated with at least X Gy (RBE).

### Statistics

Differences in the values between two groups were examined using Wilcoxon rank-sum test. The trend in skin dose reduction by LSI for S10 through S60 was examined using the Jonckheere-Terpstra test. A *P* < 0.05 was considered statistically significant. All statistical analyses were performed using SPSS (version 25; SPSS Inc., Chicago, IL, USA).

## Results

### Comparison of Target Volume Coverage

First, we compared target volume coverage between CPI and LSI in the same set of 21 parotid tumors (Table 2).

**Table 2:** Target volume coverage by conventional passive irradiation and layer-stacking irradiation.

**Target volume**	**Index**	**Conventional (mean ± SD)**	**Layer-stacking (mean ± SD)**	***P*-values**	**% difference (mean ± SD)**
CTV1	D2%	65.0 ± 0.5	64.6 ± 1.3	0.247	1.0 ± 1.9
	D50%	63.2 ± 2.1	62.4 ± 2.0	<0.001	1.2 ± 1.4
	D98%	50.5 ± 7.9	49.7 ± 7.2	0.006	2.6 ± 2.5
	HI	0.23 ± 0.12	0.24 ± 0.11	0.025	NA
CTV2	D2%	65.0 ± 0.5	65.0 ± 0.6	0.506	0.56 ± 0.59
	D50%	64.1 ± 0.5	63.8 ± 0.4	0.002	0.69 ± 0.45
	D98%	61.0 ± 3.1	60.3 ± 2.7	0.002	1.6 ± 1.0
	HI	0.06 ± 0.04	0.07 ± 0.04	<0.001	NA

*D2%, D50%, and D98% are shown in Gy (RBE). P-values were assessed by Wilcoxon rank-sum test. The % difference indicates the ratio of absolute difference between DX% for conventional passive irradiation and that for layer-stacking irradiation to DX% for conventional passive irradiation expressed as a percentage. SD, standard deviation; NA, not assessed*.

For CTV1, there were no significant differences in D2% between the two methods. D50% and D98% were significantly higher for CPI. However, the absolute differences between the two methods were small (within 2 and 3% for D50% and D98%, respectively). HI was significantly and slightly higher for LSI.

For CTV2, there were no significant differences in D2% between the two methods. D50% and D98% were significantly higher for CPI. However, the absolute differences between the two methods were small (within 1 and 2% for D50% and D98%, respectively). HI was significantly and slightly higher for LSI.

Taken together, these data indicate that target volume coverage achieved by LSI is comparable to that achieved by CPI.

### Comparison of Skin Dose

**Figure 2:**
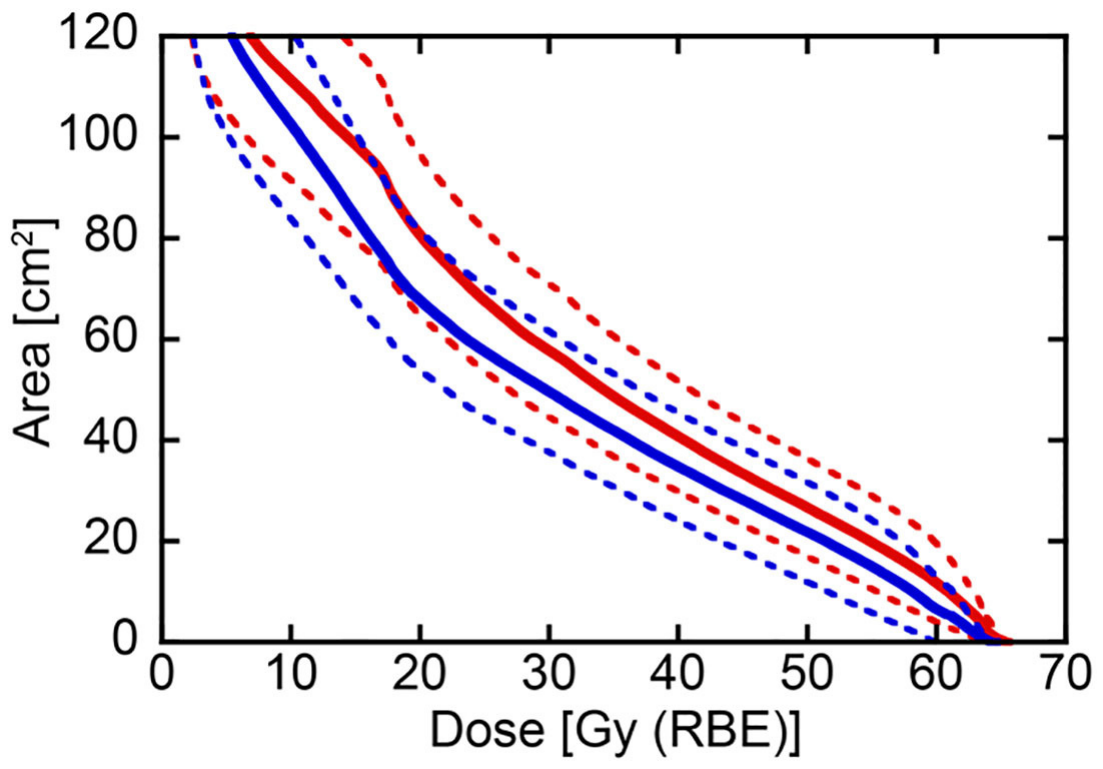
Dose-surface area histogram for the skin comparing conventional passive irradiation and layer-stacking irradiation in the same 21 parotid tumor cases. Solid and dotted lines indicate mean and 95% confidence interval, respectively. Red and blue lines show conventional passive irradiation and layer-stacking irradiation, respectively.

**Figure 3:**
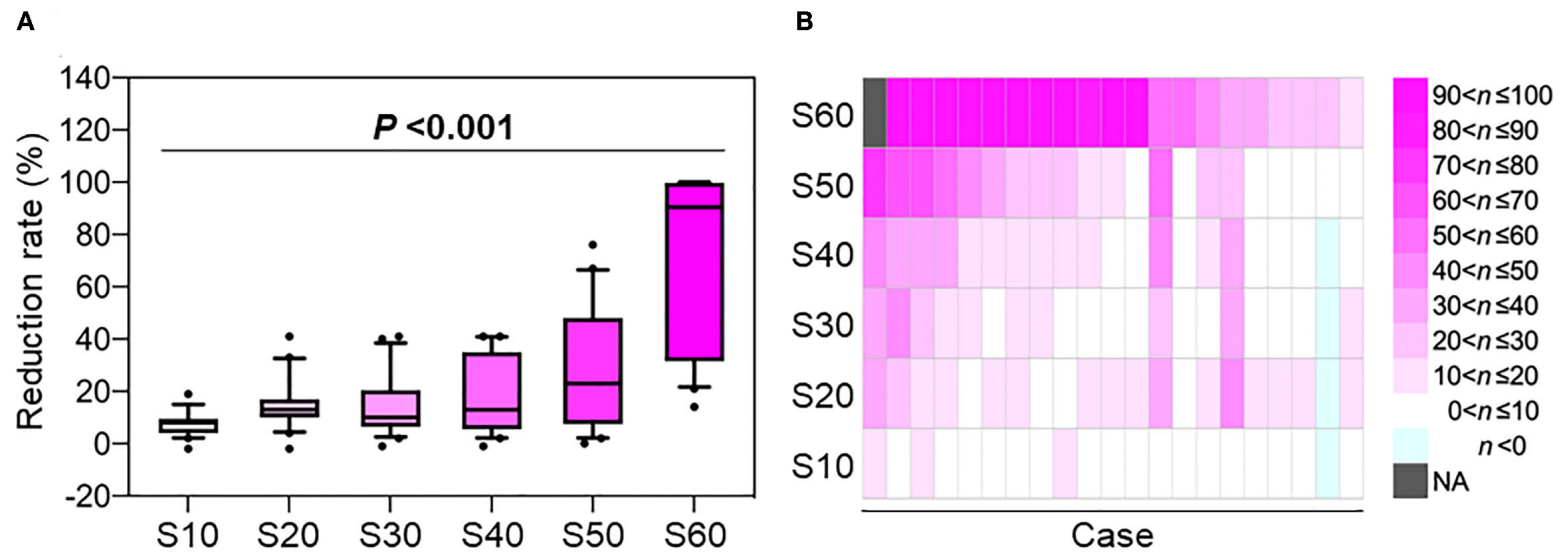
The rate of skin dose reduction in the treatment plans created using layer-stacking irradiation compared with those created by conventional passive irradiation as controls. **(A)** Box whisker plot. Boxes indicate 25th percentile through 75th percentile. Top and bottom whiskers show 10th and 90th percentile, respectively. *P-*value was assessed using the Jonckheere-Terpstra test. **(B)** Heatmap showing the data from an individual subject. *n* indicates the reduction rate expressed as a percentage. NA, not assessed because the control value was zero.

**Figure 4:**
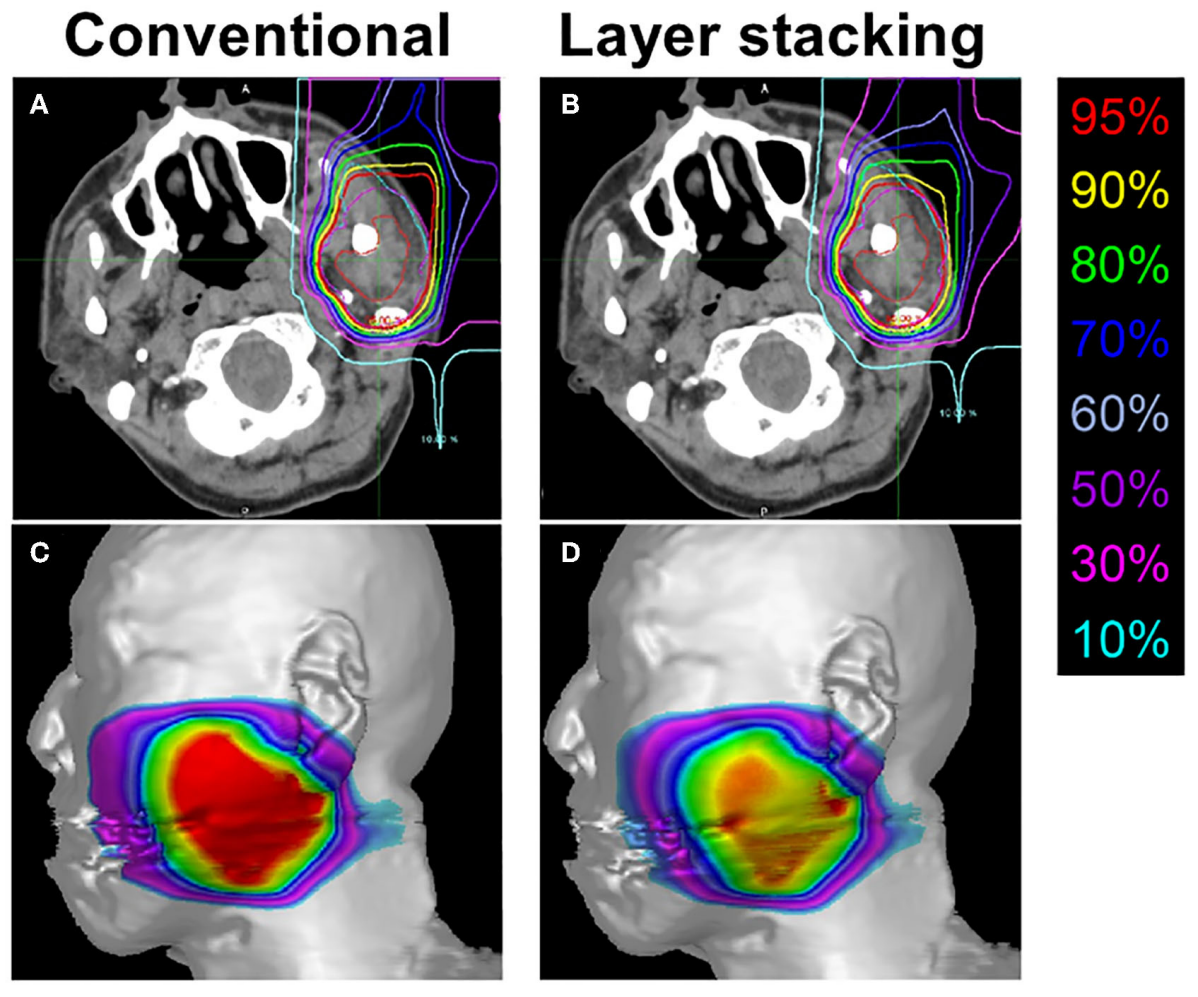
Representative treatment plans created by conventional passive irradiation and layer-stacking irradiation for the same subject. **(A,B)** Dose distribution in axial computed tomography images. Gross tumor volume, clinical target volume (CTV) 1, and CTV2 are indicated in red, cyan, and magenta, respectively; **(C,D)** dose-surface area model for the skin.

**Figure 5:**
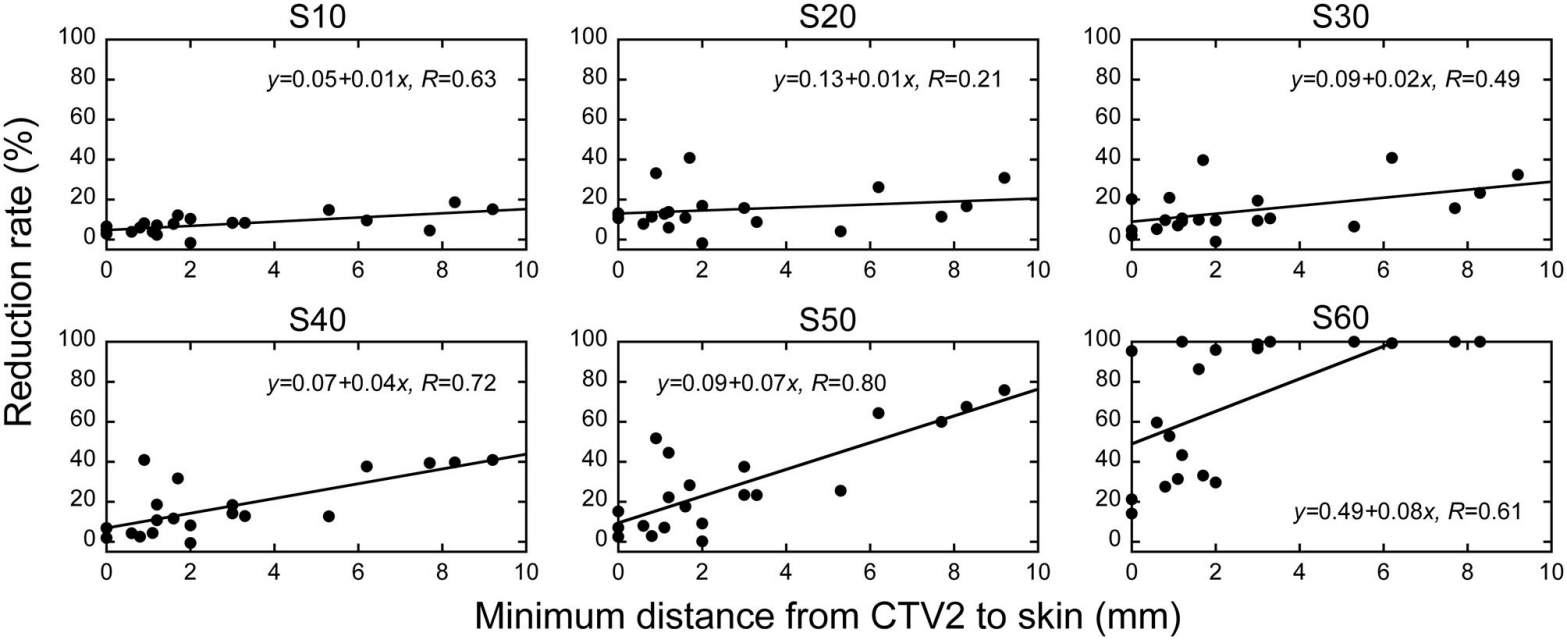
The association between the distance from CTV2 to skin at the axial isocenter sleeve and skin dose reduction rate in layer-stacking irradiation and conventional passive irradiation. The vertical axis shows the reduction in the dose of irradiation the skin receives when layer-stacking irradiation is employed. SX indicates the skin surface area irradiated with at least X Gy (RBE).

After confirming that target volume coverage was comparable between the treatment plans created using two methods, we compared the skin doses. Overall, the skin doses were lower for LSI than for CPI throughout the dose range (Figure 2). S10, S20, S30, S40, S50, and S60 were significantly lower for LSI than for CPI (Table 3). There was a significant trend toward dose reduction associated with LSI at the skin area irradiated with a higher dose by CPI (*P* < 0.001; Figures 3, 4). Taken together, these data demonstrate that skin sparing by LSI is superior to that of CPI in the treatment of parotid tumors, especially in the high-dose range. Figure 5 shows that the skin sparing ability of LSI correlated with the distance from CTV2 to the skin.

**Table 3:** Skin surface dose for conventional passive irradiation and layer-stacking irradiation.

**Index**	**Conventional (mean ± SD)**	**Layer-stacking (mean ± SD)**	***P*-values**
S10	111.3 ± 47.3	102.5 ± 44.6	<0.001
S20	80.6 ± 37.9	67.6 ± 33.1	<0.001
S30	57.7 ± 31.5	49.6 ± 28.6	<0.001
S40	40.7 ± 26.1	34.7 ± 25.5	<0.001
S50	26.6 ± 23.2	21.7 ± 23.6	<0.001
S60	11.5 ± 18.5	6.5 ± 15.4	<0.001

*P-values were assessed by Wilcoxon rank-sum test. SD, standard deviation*.

## Discussion

This is the first study comparing LSI with CPI using a cohort of patients treated with carbon ion radiotherapy. The treatment plans were tested in 21 patients with parotid tumors, and the results showed that LSI was superior to CPI regarding skin sparing, especially at the high-dose range, without compromising target volume coverage. The treatment for head-and-neck non-squamous cell carcinoma has not been standardized, and evidence suggests that carbon ion radiotherapy achieves favorable local control and overall survival in patients with this disease (13–17). Taken together, the present data suggest that carbon ion radiotherapy for head-and-neck non-squamous cell carcinomas can be improved by using LSI.

The dosimetric parameters associated with the risk of skin toxicities after carbon ion radiotherapy have been reported extensively. Takakusagi et al. reported the outcomes of malignant bone and soft tissue tumors treated with carbon ion radiotherapy and showed that grade-2 acute dermatitis increased when S40 exceeded 25 cm^2^ (12). In this study, LSI decreased the number of patients in which the S40 exceeded 25 cm^2^ by 14% (from 15 patients to 12 patients). Yanagi et al. reported the outcomes of bone and soft tissue sarcomas treated with carbon ion radiotherapy and showed that grade-3 chronic dermatitis increased when S60 exceeded 20 cm^2^ (18). In this study, LSI decreased the number of patients in which the S60 exceeded 20 cm^2^ by 33% (from 3 patients to 2 patients). The two studies by Takakusagi et al. and Yanagi et al. suggest that the risk of skin toxicities after carbon ion radiotherapy is higher in the high-dose range (i.e., S40–S60). In this study, the skin dose reduction by LSI was greater at the high-dose range. This indicates the potential of LSI for the efficient reduction of skin toxicities associated with carbon ion radiotherapy, which may improve the quality of life of patients. Further study is warranted to investigate whether skin dose reduction by CPI affects clinical outcomes.

However, LSI has several shortcomings. In the LSI systems used in our institution (i.e., GHMC) and in the National Institutes of Radiological Sciences, Japan (2), the initial MLC shape is fixed within a depth of 30 mm (i.e., 12 steps). Therefore, achieving dose distribution conformal to the tumors using LSI is difficult when the tumor diameter is <30 mm. In the present cohort, the LSI-based treatment plan resulted in a slightly higher skin dose than that of the CPI-based treatment plan in a patient with a small tumor whose CTV2 volume was 10.1 cm^3^ (as indicated in light blue in the second case from the right in Figure 3). In addition, irradiation time is longer for LSI than for CPI. In the present study, the median irradiation times per port for CPI and LSI were 46 and 105 s, respectively. Therefore, the indications for LSI should be carefully determined according to tumor size.

The present study had several limitations. First, the skin dose was analyzed in a relatively small number of parotid tumor cases (*n* = 21). Second, the effect of LSI on dose reduction in other organs at risk needs to be investigated in cancers other than parotid tumors. Further studies using larger cohorts would help identify the patients who would most benefit from LSI.

In summary, we showed, for the first time, that LSI is superior to CPI regarding skin sparing, especially at the high-dose range, without sacrificing target volume coverage in patients with parotid tumors. Further studies are warranted to determine the benefits of LSI for other cancers and other organs at risk.

## Data Availability Statement

The raw data supporting the conclusions of this article will be made available by the authors, without undue reservation, to any qualified researcher.

## Ethics Statement

The studies involving human participants were reviewed and approved by the institutional review board of Gunma University Hospital. Written informed consent for participation was not required for this study in accordance with the national legislation and the institutional requirements.

## Author Contributions

NK, YK, MS, and TOi designed and directed the analyses. NK, YK, AI, SK, YM, HS, AM, and NO generated a database and performed data collection. NK, YK, and TOi participated substantially in the preparation of the manuscript. HK, KS, JS, KC, and TOh supervised the project. All authors provide approval for publication of the content.

## Conflict of Interest

The authors declare that the research was conducted in the absence of any commercial or financial relationships that could be construed as a potential conflict of interest.
